# Maternal Adenine-Induced Chronic Kidney Disease Programs Hypertension in Adult Male Rat Offspring: Implications of Nitric Oxide and Gut Microbiome Derived Metabolites

**DOI:** 10.3390/ijms21197237

**Published:** 2020-09-30

**Authors:** Chien-Ning Hsu, Hung-Wei Yang, Chih-Yao Hou, Guo-Ping Chang-Chien, Sufan Lin, You-Lin Tain

**Affiliations:** 1 Department of Pharmacy, Kaohsiung Chang Gung Memorial Hospital, Kaohsiung 833, Taiwan; cnhsu@cgmh.org.tw; 2 School of Pharmacy, Kaohsiung Medical University, Kaohsiung 807, Taiwan; 3 Institute of Medical Science and Technology, National Sun Yat-sen University, Kaohsiung 804, Taiwan; howardyang@imst.nsysu.edu.tw; 4 Department of Seafood Science, National Kaohsiung University of Science and Technology, Kaohsiung 811, Taiwan; chihyaohou@webmail.nkmu.edu.tw; 5 Center for Environmental Toxin and Emerging-Contaminant Research, Cheng Shiu University, Kaohsiung 833, Taiwan; guoping@csu.edu.tw (G.-P.C.-C.); linsufan2003@csu.edu.tw (S.L.); 6 Super Micro Mass Research and Technology Center, Cheng Shiu University, Kaohsiung 833, Taiwan; 7 Department of Pediatrics, Kaohsiung Chang Gung Memorial Hospital and College of Medicine, Chang Gung University, Kaohsiung 833, Taiwan; 8 Institute for Translational Research in Biomedicine, College of Medicine, Kaohsiung Chang Gung Memorial Hospital and Chang Gung University, Kaohsiung 833, Taiwan

**Keywords:** asymmetric dimethylarginine, chronic kidney disease, developmental origins of adult health and disease (DOHaD), gut microbiota, hypertension, nitric oxide, renin-angiotensin system, short chain fatty acid, trimethylamine-*N*-oxide, uremic toxin

## Abstract

Maternal chronic kidney disease (CKD) during pregnancy causes adverse fetal programming. Nitric oxide (NO) deficiency, gut microbiota dysbiosis, and dysregulated renin-angiotensin system (RAS) during pregnancy are linked to the development of hypertension in adult offspring. We examined whether maternal adenine-induced CKD can program hypertension and kidney disease in adult male offspring. We also aimed to identify potential mechanisms, including alterations of gut microbiota composition, increased trimethylamine-*N*-oxide (TMAO), reduced NO bioavailability, and dysregulation of the RAS. To construct a maternal CKD model, female Sprague-Dawley rats received regular chow (control group) or chow supplemented with 0.5% adenine (CKD group) for 3 weeks before pregnancy. Mother rats were sacrificed on gestational day 21 to analyze placentas and fetuses. Male offspring (*n* = 8/group) were sacrificed at 12 weeks of age. Adenine-fed rats developed renal dysfunction, glomerular and tubulointerstitial damage, hypertension, placental abnormalities, and reduced fetal weights. Additionally, maternal adenine-induced CKD caused hypertension and renal hypertrophy in adult male offspring. These adverse pregnancy and offspring outcomes are associated with alterations of gut microbiota composition, increased uremic toxin asymmetric and symmetric dimethylarginine (ADMA and SDMA), increased microbiota-derived uremic toxin TMAO, reduced microbiota-derived metabolite acetate and butyrate levels, and dysregulation of the intrarenal RAS. Our results indicated that adenine-induced maternal CKD could be an appropriate model for studying uremia-related adverse pregnancy and offspring outcomes. Targeting NO pathway, microbiota metabolite TMAO, and the RAS might be potential therapeutic strategies to improve maternal CKD-induced adverse pregnancy and offspring outcomes.

## 1. Introduction

Chronic kidney disease (CKD) is a highly prevalent disease worldwide, as at least 3–4% of childbearing-aged women’s lives are complicated by this condition [1,2]. In utero, environmental factors such as maternal illness play a key role in the developmental programming of kidney disease and hypertension [3,4]. Current evidence provides the notion that many adult diseases can originate in early life [5]. Although most studies demonstrated that pregnant women with CKD are at risk of adverse maternal and perinatal outcomes [6,7], less attention has been paid to exploring the effects of maternal CKD on renal outcomes in adult offspring.

Disturbance of normal gut microbiota could link early-life environmental insults to later risk of adult diseases [8]. Recent studies have identified several possible interconnected mechanisms between gut dysbiosis and hypertension [9,10], such as altered microbial composition, increased production of microbiota-derived trimethylamine-*N*-oxide (TMAO), changes of short chain fatty acids (SCFAs) and their receptors, impaired nitric oxide (NO) pathway, and dysregulation of the renin-angiotensin system (RAS). In CKD, uremic toxin accumulation plays a crucial role in cardiovascular risk. In addition to microbiota-related TMAO [11], asymmetric and symmetric dimethylarginine (ADMA and SDMA, endogenous inhibitors of NO synthase) are major uremic toxins that contribute to the pathogenesis of cardiovascular disease [12].

We previously reported that offspring hypertension programmed by various maternal insults is related to NO deficiency [13], alterations of gut microbiota composition and microbial metabolite TMAO [14], and dysregulation of the RAS [15], all of which are decisive mechanisms underlying hypertension. However, whether the above-mentioned mechanisms interconnect to maternal CKD-induced offspring hypertension and kidney disease of developmental origins is still largely unknown.

The adenine diet model results in progressive kidney injury with cardiovascular changes, faithfully mimicking the pathophysiology of human CKD [16,17]. The aim of our work was to determine whether maternal adenine-induced CKD can program hypertension and kidney disease in adult offspring and whether it is associated with altered gut microbiota composition, reduced NO, increased TMAO, and dysregulated RAS.

## 2. Results

### 2.1. Renal Outcome in Mother Rats

**Figure 1:**
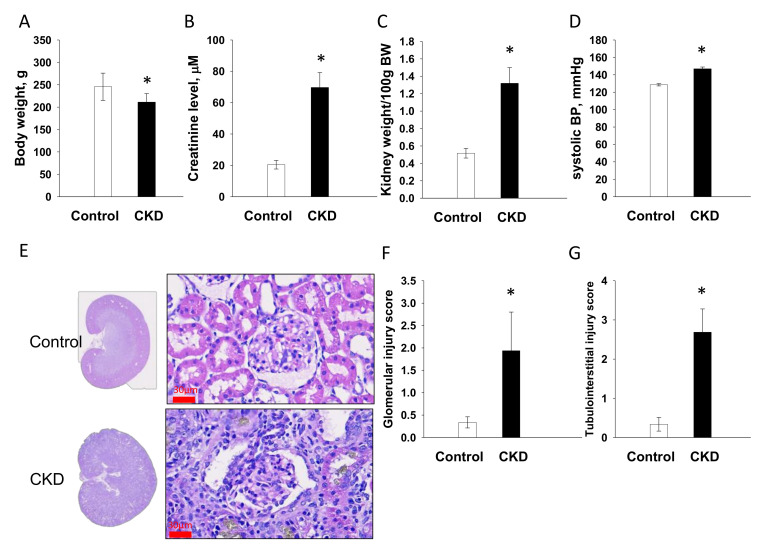
Renal outcome measures in mother rats. (**A**) Body weights, (**B**) plasma creatinine level, (**C**) kidney weight-to-body weight ratio, (**D**) systolic blood pressures, (**E**) periodic acid-Schiff stained sections demonstrating renal histopathology of female rats fed with regular chow (control) or chow supplemented with 0.5% adenine (chronic kidney disease, CKD) for 3 weeks before pregnancy; scale bar = 30 μm, (**F**) glomerular injury score, and (**G**) tubulointerstitial injury score. Data are shown as means ± SD; *n* = 8/group. The asterisk indicates *p* < 0.05 vs. control.

Figure 1 displays adenine-treated mother rats had lower body weights (BWs) (Figure 1A), higher creatinine levels (Figure 1B), higher kidney weight-to-BW ratios (Figure 1C), and higher systolic blood pressures (SBP, Figure 1D) compared to the controls. Consistent with previous reports [16,17], the kidneys of adenine-fed rats were grossly enlarged with segmental necrosis, thickening in the basal membrane of glomeruli and tubules, interstitial inflammatory infiltrates, adenine crystalline deposits, tubular dilatation, and tubular atrophy (Figure 1E). Adenine-treated CKD group had greater degrees of glomerular (Figure 1F) and tubulointerstitial injury (Figure 1G) than controls. These data indicate that mother rats developed CKD before pregnancy, which is characterized by impaired renal function (~30% of normal renal function), renal hypertrophy, glomerular and tubulointerstitial injuries, and hypertension.

### 2.2. Placenta and Fetal Weight

**Figure 2:**
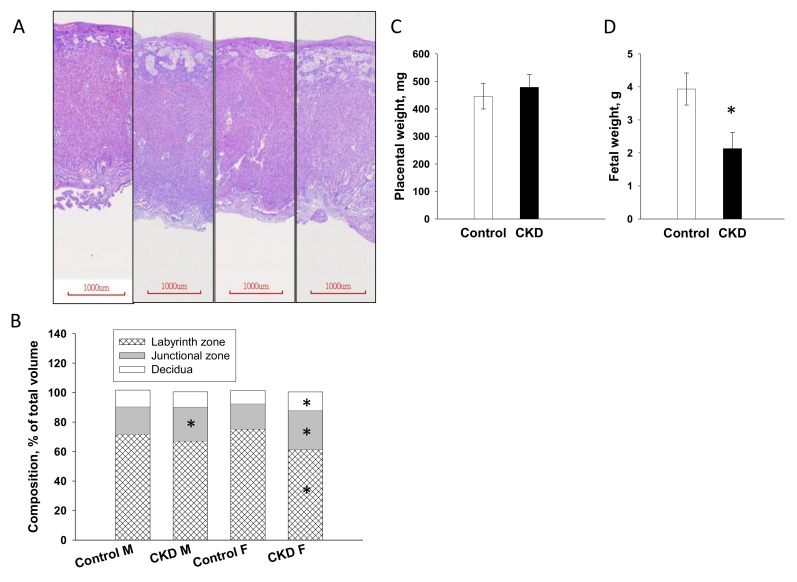
Placenta and fetal weight. (**A**) Hematoxylin and eosin stained sections demonstrating histological appearance of the placental layers of pregnant rats fed with regular chow (control) or chow supplemented with 0.5% adenine (CKD) at gestational day 21; scale bar = 1000 μm, (**B**) mean percentage of total volume of the decidua, junctional zone, and labyrinth zone; M = male fetus; F = female fetus, (**C**) placental weight, and (**D**) fetal weight. Data are shown as means ± SD; *n* = 5/group. The asterisk indicates *p* < 0.05 vs. control.

As presented in Figure 2, maternal CKD caused a significant increase in thickness and volume of the junctional zone in male placenta. In female placenta from CKD dams, volume of the junctional zone was lower, whereas labyrinth and decidua layers were higher than those in female control placenta (Figure 2B). Maternal CKD had a negligible effect on litter sizes (pups per litter: control vs. CKD = 16 ± 2.2 vs. 11 ± 1.4, *p* = 0.052) and sex ratio (M/F ratio: control vs. CKD = 50% vs. 57.6%). Placental weights were not different between the two groups (Figure 2C), while maternal CKD significantly reduced fetal weights (Figure 2D).

### 2.3. Gut Microbiota Composition in Mother Rats

**Figure 3:**
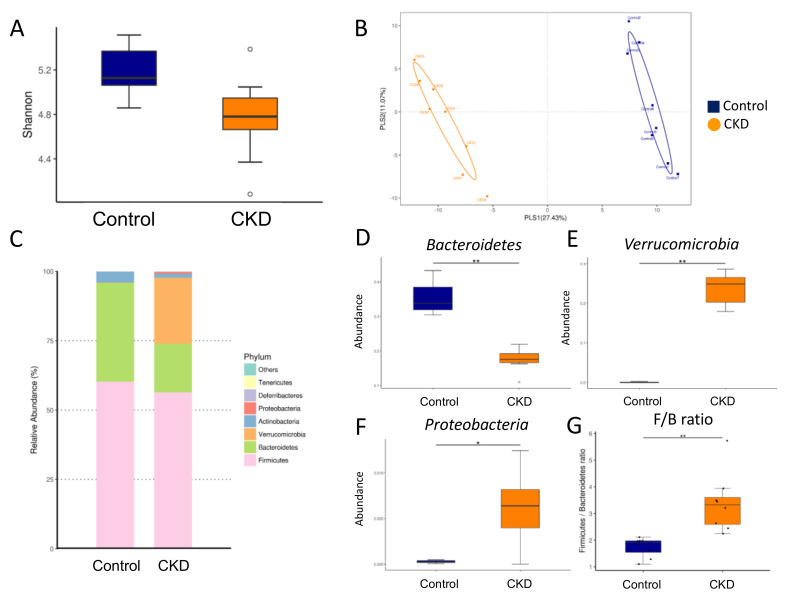
Gut microbiota at the phylum level in mother rats. (**A**) Variation in fecal bacterial α-diversity represented by the Shannon’s diversity indexes, (**B**) β-diversity changes in gut microbiota between the two groups by the partial least squares discriminant analysis (PLS-DA), (**C**) relative abundance of top 10 phyla of the gut microbiota between the two groups. The abundance of phyla (**D**) *Bacteroidetes*, (**E**) *Verrucomicrobia*, and (**F**) *Patescibacteria* between the two groups. (**G**) The *Firmicutes* to *Bacteroidetes* ratio between the two groups. Control = female rats fed with regular chow; CKD = female rats fed with chow supplemented with 0.5% adenine for 3 weeks before pregnancy. Abundance = the number of bacterial operational taxonomic unit (OTU) sequences detected in each sample. Data are shown as means ± SD; *n* = 8/group. The asterisk indicates *p* < 0.05 vs. control. The double asterisk indicates *p* < 0.01 vs. control.

We next compared the difference in gut microbiota among the adenine-induced CKD mother rats and controls. Microbiome diversity is typically defined in terms of within (i.e., α-diversity) and between community/sample (i.e., β-diversity) diversities [18]. The Shannon diversity index, a commonly used α-diversity measure to determine how evenly the microbes are distributed in a community, was not statistically different between two groups (Figure 3A). In the current study, two different β-diversity analysis techniques were performed to compare the bacterial community similarity, including the partial least squares discriminant analysis (PLS-DA) and the analysis of similarities (ANOSIM). The scatterplots of PLS-DA analysis demonstrated that the CKD group and the control group were well separated (Figure 3B), which was supported by ANOSIM analysis showing a significant difference between the two groups (*p* = 0.001). These findings indicated that both groups had distinct enterotypes. The major bacteria phyla found in the present study were *Firmicutes*, *Bacteroidetes*, *Verrucomicrobia*, *Actinobacteria*, and *Patescibacteria* (Figure 3C). Adenine-induced CKD caused a remarkable decrease in the phylum *Bacteroidetes* (*p* < 0.001) (Figure 3D) but an increase in the *Verrucomicrobia* (Figure 3E, *p* < 0.001) and the *Patescibacteria* (Figure 3F, *p* = 0.034). The *Firmicutes* to *Bacteroidetes* ratio has been considered a signature for hypertension [10]. Our data showed that the *Firmicutes* to *Bacteroidetes* ratio was higher in the CKD group compared to the controls (Figure 3G, *p* = 0.001).

**Figure 4:**
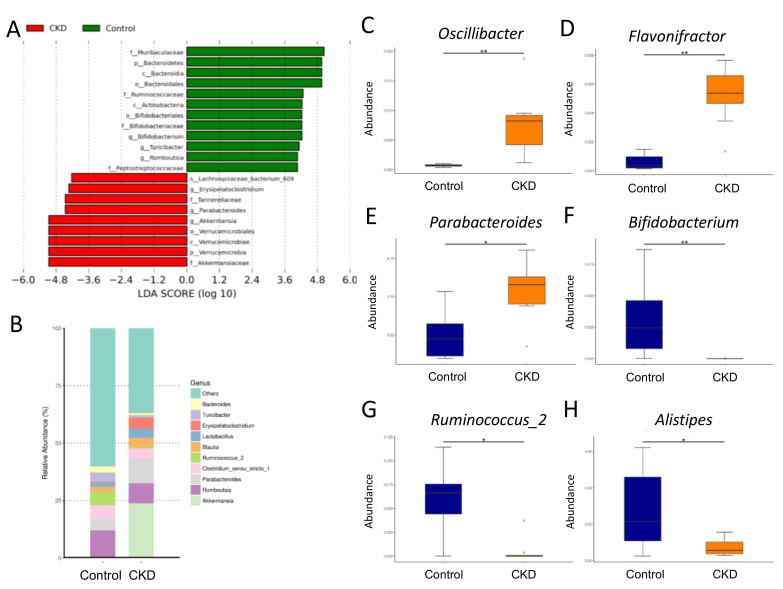
Gut microbiota at the genus level in mother rats. (**A**) The linear discriminant analysis effect size (LEfSe) to identify the taxa that were significantly different between the two groups. (**B**) The relative abundance of top 10 genera of the gut microbiota between the two groups. The abundance of genera (**C**) *Oscillibacter*, (**D**) *Flavonifractor*, (**E**) *Parabacteroides*, (**F**) *Bifidobacterium*, (**G**) *Ruminococcus_2*, and (**H**) *Alistipes* between the two groups. Control = female rats fed with regular chow; CKD = female rats fed with chow supplemented with 0.5% adenine for 3 weeks before pregnancy. Abundance = the number of bacterial operational taxonomic unit (OTU) sequences detected in each sample. Data are shown as means ± SD; *n* = 8/group. The asterisk indicates *p* < 0.05 vs. control. The double asterisk indicates *p* < 0.01 vs. control.

We performed linear discriminant analysis effect size (LEfSe) analysis to identify differentially over or under-represented taxa between the groups (Figure 4A). The LEfSe analysis identified a higher abundance of phylum *Verrucomicrobia* and a lower abundance of phylum *Bacteroidetes* in the CKD group vs. the controls. At the genus level, the major genera were similar between the two groups (Figure 4B). We observed that adenine-induced CKD increased the abundance of the genera *Oscillibacter* (Figure 4C, *p* < 0.001), *Flavonifractor* (Figure 4D, *p* < 0.001), and *Parabacteroides* (Figure 4E, *p* = 0.012). Conversely, the abundances of the genera *Bifidobacterium* (Figure 4F, *p* < 0.001), *Ruminococcus_2* (Figure 4G, *p* = 0.014), and *Alistipes* (Figure 4H, *p* = 0.032) were lower in the CKD group compared to the controls.

### 2.4. NO Pathway

We next investigated whether adenine-induced CKD impairs the NO pathway (Table 1). Plasma levels of L-citrulline, ADMA, and SDMA were significantly increased, while L-arginine was decreased in the CKD group in comparison with the controls. Additionally, we found that adenine-induced CKD significantly reduced plasma L-arginine-to-ADMA ratio, an index of NO bioavailability, in CKD rats vs. the controls.

### 2.5. TMAO Pathway

Like ADMA and SDMA, TMAO is a uremic toxin correlated with hypertension. Trimethylamine (TMA) is a precursor of TMAO. Both TMA and TMAO can be metabolized to dimethylamine (DMA). We, therefore, simultaneously determined TMAO, TMA, and DMA concentrations in the plasma (Table 1). Adenine-induced CKD significantly increased plasma TMAO and DMA levels. Plasma TMA level was comparable between two groups. These findings strongly suggest that the TMA-TMAO metabolic pathway is augmented in CKD mother rats.

### 2.6. Short Chain Fatty Acids in Plasma and Feces

Because SCFAs are important microbiota-derived metabolites and certain SCFAs are related to the development of hypertension, we subsequently investigated three major SCFAs, acetate, propionate, and butyrate, in the plasma and the feces. As shown in Table 2, plasma acetate and butyrate levels were lower in the CKD rats than those in the controls. In feces, adenine-induced CKD significantly reduced acetate and propionate concentrations.

### 2.7. Renal Outcome in Offspring

**Figure 5:**
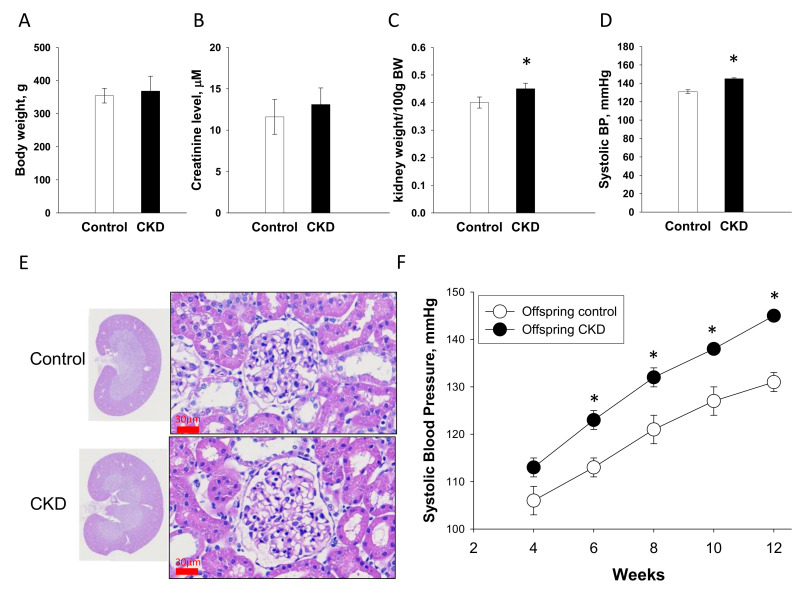
Renal outcome measures in male offspring rats. (**A**) Body weights, (**B**) plasma creatinine level, (**C**) kidney weight-to-body weight ratio, (**D**) systolic blood pressures, and (**E**) periodic acid-Schiff stained sections demonstrating renal histopathology of male offspring rats at 12 weeks of age; scale bar = 30 μm, (**F**) systolic blood pressure in male offspring from 4 to 12 weeks of age. Control = male offspring born to dams fed with regular chow; CKD = male offspring born to dams received chow supplemented with 0.5% adenine for 3 weeks before pregnancy. Data are shown as means ± SD; *n* = 8/group. The asterisk indicates *p* < 0.05 vs. control.

The renal outcomes in 12-week-old male offspring are reported in Figure 5. We found mortality rates, BW (Figure 5A), and plasma creatinine level (Figure 5B) did not differ between the two groups. Maternal CKD significantly increased kidney weight-to-BW ratios (Figure 5C) and SBP (Figure 5D) in the male offspring than in the controls. The kidney sections of the offspring rats in the control and the CKD groups generally displayed normal histological structure (Figure 5E). As illustrated in Figure 5F, SBP was comparable between two groups at 4 weeks of age. From 6 to 12 weeks of age, SBP was significantly increased in CKD offspring compared with that in control offspring. These data indicate that maternal CKD causes renal hypertrophy and hypertension in adult male offspring.

### 2.8. NO Pathway and the RAS in Offspring

**Figure 6:**
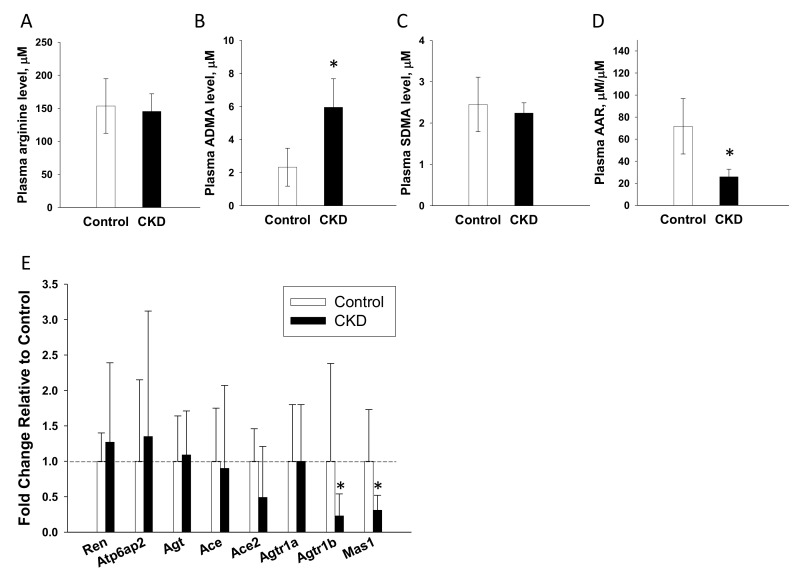
Nitric oxide (NO) system and renin-angiotensin system (RAS) in male offspring rats. (**A**) Plasma L-arginine level, (**B**) plasma asymmetric dimethylarginine (ADMA) level, (**C**) plasma symmetric dimethylarginine (SDMA) level, and (**D**) L-arginine-to-ADMA ratio (AAR) between the two groups. (**E**) Renal mRNA expression of the RAS components between the two groups. Control = male offspring born to dams fed with regular chow; CKD = male offspring born to dams received chow supplemented with 0.5% adenine for 3 weeks before pregnancy. *Ren* = renin; *Atp6ap2* = prorenin receptor; *Agt* = angiotensinogen, *Ace* = angiotensin converting enzyme-1; *Ace2* e = angiotensin converting enzyme-2; *Agtr1a =* angiotensin II type 1 receptor; *Agtr1b* = angiotensin II type 2 receptor, *Mas1* = angiotensin (1–7) receptor. Data are shown as means ± SD; *n* = 8/group. The asterisk indicates *p* < 0.05 vs. control.

To further investigate mechanisms underlying programmed hypertension in adult offspring, we evaluated the NO-related parameters and the renal mRNA expression of RAS components (Figure 6). Our results demonstrated that CKD offspring had higher plasma ADMA level (Figure 6B) and lower L-arginine-to-ADMA ratio (Figure 6D) compared with control offspring. Additionally, renal mRNA expressions of Agtr1b and Mas1 were lower in the CKD vs. the control offspring (Figure 6E).

## 3. Discussion

This study provides a new insight into the mechanisms underlying maternal CKD-induced hypertension in adult male offspring with particular emphasis on gut microbiota metabolites, NO pathway, and the RAS. Our major findings in this study are: (1) the adenine-induced maternal CKD is a useful experimental model to study the effects of uremia on pregnancy and offspring outcomes; (2) adenine-induced CKD is associated with alterations of gut microbiota composition, increased plasma TMAO and DMA levels, reduced plasma acetate and butyrate levels, impaired NO pathway, and dysregulated RAS; (3) adenine-induced CKD dams display increased placental junctional zone and reduced fetal weights; and (4) maternal CKD increases risks of adverse renal outcomes in adult male offspring, including hypertension and renal hypertrophy.

In line with previous studies demonstrating adenine-induced CKD model mimics the complexity of the human CKD [16,17,19], adenine-fed mother rats exhibited renal dysfunction, glomerular and tubulointerstitial damage, hypertension, and increased uremic toxin levels. This model also allows the characterization of placental abnormalities and intrauterine growth retardation (IUGR), similar to adverse pregnancy outcomes in women with CKD [1,2,6,7]. Our results go beyond previous reports, showing that maternal CKD induced hypertension and renal hypertrophy in adult male offspring. Although renal function in adult offspring remains intact, renal hypertrophy, a characteristic early feature of kidney disease, has appeared. By providing common features of human CKD, the adenine-induced maternal CKD may be a suitable experimental model for studying the therapeutic interventions to protect against uremia-related adverse pregnancy and offspring outcomes in humans.

In support of the notion that uremic toxins contribute to hypertension in CKD [12,20], our results demonstrated that maternal adenine-induced CKD mother rats exhibited hypertension related to elevated plasma levels of ADMA, SDMA, and TMAO. ADMA and SDMA are both uremic toxins, and their most well-known effect is the suppression of NO production [12]. A disturbed ADMA-NO balance is involved in compromised pregnancy, adverse fetal programming, and hypertension [13,21,22]. We observed adenine-induced CKD dams developed hypertension was associated with increased ADMA and SDMA but decreased L-arginine-to-ADMA ratio, an index of NO bioavailability [23]. Likewise, CKD offspring developed hypertension related to elevated ADMA levels and reduced L-arginine-to-ADMA ratios in the plasma. NO deficiency has been reported to be involved in CKD and hypertension [24,25]. In the current study, we provide further evidence for an association between maternal CKD-induced offspring hypertension and NO deficiency. We and others have shown that an early restoration of ADMA-NO balance protects against the development of hypertension in later life [22,26]. Further investigation should be warranted to evaluate the potential beneficial effects of early NO therapy on offspring hypertension programmed by maternal CKD.

Additionally, the detrimental effects of maternal CKD on adverse offspring’s renal outcomes might be owing to increased TMAO, a microbiota-derived uremic toxin. Results from this study identified plasma TMAO level is increased in adenine-induced CKD mother rats, which is in accordance with data from uremic patients [11]. According to our data, the abundance of *Proteobacteria*, the main phylum in the TMA-producing community [27], was higher in the CKD group. Likewise, adenine-induced CKD resulted in increases of several bacteria known to be associated with formation of TMA [28], including genera *Oscillibacter*, *Flavonifractor*, and *Parabacteroides*. These findings support the link between these TMA-TMAO metabolizing microbes, CKD, and microbiota-derived uremic toxins. Consistent with prior research in humans with CKD [10,29,30], low abundance of genera *Bifidobacterium*, *Ruminococcus*, and *Alistipes*, whereas high abundance of genus *Oscillibacter* were identified as microbial markers in this adenine-induced CKD model. Moreover, our data showed adenine-induced CKD mother rats displayed hypertension was associated with a high ratio of *Firmicutes* to *Bacteroidetes*, low abundance of genera *Bifidobacterium* and *Alistipes*, and decreased plasma acetate and butyrate levels. Our results tie well with those of previous studies demonstrating that alterations of specific gut microbial composition and metabolites have an essential role in hypertension development [9,10]. Given that inhibition of the TMA-TMAO metabolic pathway has been reported to protect adult offspring against hypertension programmed by early life insult and CKD progression [31,32,33], outcomes from targeting the TMA-TMAO pathway to protect against CKD-related adverse pregnancy and offspring need further research.

In addition to TMAO, current evidence supports that gut microbiota derived SCFAs are also involved in CKD and BP regulation [10,34]. Consistent with previous reports showing decreased levels of SCFAs in CKD [10,35], we observed that adenine-induced CKD reduced fecal acetate and propionate as well as plasma acetate and butyrate concentrations. Since acetate and propionate have been reported to induce vasodilatation via mediating the SCFA receptor [34], reduced levels of SCFAs are presumed to elevate BP. We and others have shown beneficial effects of acetate supplementation on CKD and hypertension of developmental origin [31,36]. It is interesting to elucidate whether maternal SCFA supplementation could exert benefits on offspring hypertension programmed by maternal CKD.

Another harmful effect of maternal CKD on offspring hypertension could be due to dysregulation of the RAS. We found maternal CKD reduced *Agtr1b* and *Mas1* expression in offspring kidneys, which appear to be correlated with the increases of SBPs in adult offspring. It is well-established that *Agtr1b* and *Mas1*, belonging to the non-classical RAS, represent an endogenous counter-regulatory pathway, the actions of which are in opposition to the classical RAS axis [37]. Our data suggest that lack of protective arms of the non-classical RAS promotes vasoconstriction and elevates BP in adult offspring. Our previous studies demonstrated that maternal high-fat diet induced IUGR and increased risk for developing hypertension and kidney disease in adult offspring, which are related to altered placental morphology, placental RAS activation, and dysregulated intrarenal RAS [38,39]. Since adenine-induced maternal CKD model is characterized with IUGR, placental abnormalities, and dysregulated intrarenal RAS, our findings imply that mediation of the local RAS might be a potential target against maternal CKD-induced adverse offspring outcomes.

The present study indicated that the adenine-induced maternal CKD may be a suitable experimental model to study the effects of uremia on maternal and offspring outcomes. The limitations of this study include investigation of only male offspring, the lack of long-term renal outcomes of adenine-induced mother rats, and the lack of confirmation of our findings in other models of CKD.

## 4. Materials and Methods

### 4.1. Animals and Experimental Design

The experimental protocol was approved by the Institutional Animal Ethics Committee of Chang Gung Memorial Hospital (Permit Number 2019081501) and was carried out in strict accordance with the guidelines for animal experimentation endorsed by that committee and conformed to the Guide for the Care and Use of Laboratory Animals of the National Institutes of Health. Virgin Sprague–Dawley (SD) rats purchased from BioLASCO Taiwan Co., Ltd. (Taipei, Taiwan) were used for breeding. Rats were allowed to acclimatize in light/dark cycles of 12:12 h in a temperature (22 ± 1 °C) and humidity (55 ± 5%) controlled room in a core animal facility accredited by the Association for Assessment and Accreditation of Laboratory Animal Care International. To construct a maternal CKD model, 8-week-old female SD rats received regular chow (control group) or chow supplemented with 0.5% adenine (CKD group) for 3 weeks as described previously [19,40]. At 11 weeks of age, some rats (*n* = 8/group) were sacrificed to evaluate the severity of CKD, while others were caged with male rat for mating. Feces from each rat before sacrifice was collected. Additionally, BP was measured using an indirect tail-cuff method (BP-2000; Visitech Systems, Inc., Apex, NC, USA). Heparinized blood samples and the kidneys were collected and stored at −80 °C in the freezer.

Mating was confirmed by the presence of a copulatory plug in the vaginal-cervical region. Gestational day (GD) 1 was considered to be mating day 1. Some mother rats (*n* = 3/group) were sacrificed on GD 21. Placentas and fetuses were obtained and weighed. We labeled each placenta and fetus according to position in each uterine horn. Placentas from bilateral proximal uterine horns were used for further study. Placentas were fixed in 10% formalin in neutral buffered solution for histological analysis. Other mother rats were kept until delivery. After birth, male offspring were culled to eight pups to standardize the quantity of milk and the maternal pup care received and were used in subsequent experiments. We only selected male offspring from each litter due to hypertension and kidney disease occurring at an earlier age and at a higher rate in males than females [41].

Male offspring were assigned to two experimental groups (*n* = 8/group): control offspring born to dams who received vehicle treatment and offspring born to dams with adenine-induced CKD. BP was measured in conscious rats every two weeks over the course of eight weeks using an indirect tail-cuff method (BP-2000; Visitech Systems, Inc., Apex, NC, USA). As we described previously [36], the rats were acclimated to restraint and tail-cuff inflation for 1 week prior to the measurement to ensure accuracy and reproducibility. All male offspring were sacrificed at 12 weeks of age. Blood samples were collected in heparinized tubes at the end of the study, and the kidneys were subsequently collected. Kidneys were harvested after perfusion with phosphate buffered saline, divided into the cortex and medulla regions, snap-frozen, and stored at −80 °C in the freezer.

### 4.2. High-Performance Liquid Chromatography (HPLC)

Plasma L-citrulline (a precursor of L-arginine), L-arginine (the substrate for NO synthase), ADMA, and SDMA levels were measured using HPLC (HP series 1100, Agilent Technologies, Inc., Santa Clara, CA, USA) with the O-phthalaldehyde/3-mercaptopropionic acid (OPA/3MPA) derivatization reagent as we described previously [36]. According to a protocol validated in our lab [21], plasma creatinine level was also determined by HPLC.

### 4.3. Liquid Chromatography–Mass Spectrometry (LC–MS) Analysis

We analyzed plasma levels of TMAO, TMA (the precursor of TMAO), and dimethylamine (DMA, the metabolite of TMAO and TMA) by LC–MS/MS analysis using an Agilent 6410 Series Triple Quadrupole mass spectrometer (Agilent Technologies, Wilmington, DE, USA) equipped with an electrospray ionization source, as described previously [14]. We used diethylamine as an internal standard. The multiple reaction monitoring mode was set up using characteristic precursor product ion transitions to detect *m*/*z* 76.1→58.1, *m*/*z* 60.1→44.1, and *m*/*z* 46.1→30, for TMAO, TMA, and DMA, respectively.

### 4.4. Gas Chromatography-Flame Ionization Detector (GC-FID)

Plasma and fecal acetate, propionate, and butyrate levels were measured using gas chromatography-mass spectrometry (GCMS-QP2010; Shimadzu, Kyoto, Japan) with flame ionization detector (FID). According to our validated protocol [14], dry air, nitrogen, and hydrogen were supplied to the FID at 300, 20, and 30 mL/min, respectively. An aliquot of 2 µL sample was injected into the column. The inlet and the FID temperature were set at 200 and 240 °C, respectively. The total running time was 17.5 min. Fecal concentrations of SCFAs were represented as mM/gm feces.

### 4.5. Analysis of Gut Microbiota Composition

Frozen fecal samples were analyzed with metagenomics focused on the V3-V4 of the 16S DNA gene. As described previously [14], all polymerase chain reaction amplicons were mixed together with the Biotools Co., Ltd. (Taipei, Taiwan) for sequencing using an Illumina Miseq platform (Illumina, CA, USA). The sequences were analyzed using QIIME version 1.9.1. Sequences with a distance-based similarity of 97% or greater were grouped into operational taxonomic units (OTUs) using the USEARCH algorithm. The phylogenetic relationships were determined based on a representative sequence alignment using Fast-Tree. We compared patterns of α- and β- diversity for microbial communities [18]. Shannon’s diversity index accounting for both abundance and evenness of the taxa present was analyzed by QIIME version 1.9.1. We evaluated the β-diversity changes in gut microbiota across groups by PLS-DA and ANOSIM. To determine the significantly differential taxa, LEfSe was applied to compare samples between groups. The threshold of the linear discriminant was set to 3.

### 4.6. Quantitative Real-Time Polymerase Chain Reaction (qPCR)

RNA was extracted as described previously [6]. Several components of RAS analyzed in this study included renin (*Ren*), prorenin receptor (*Atp6ap2*), angiotensinogen (*Agt*), angiotensin converting enzyme-1 (*Ace*), *Ace2*, angiotensin II type 1 receptor (*Agtr1a*), angiotensin II type 2 receptor (*Agtr1b*), and angiotensin (1–7) receptor *Mas1*. We used 18S rRNA (*r18S*) as a reference. Primers were designed using GeneTool Software (BioTools, Edmonton, AB, Canada). Table 3 shows primer sequences for qPCR. Each sample was run in duplicate. To quantify the relative gene expression, the comparative threshold cycle (C_T_) method was employed. For each sample, the average C_T_ value was subtracted from the corresponding average *Rn18s* value, calculating the ΔC_T_. ΔΔC_T_ was calculated by subtracting the average control ΔC_T_ value from the average experimental ΔC_T_. The fold-increase of the experimental sample relative to the control was calculated using the formula 2^−ΔΔCT^.

### 4.7. Histology and Morphometric Study

The formalin-fixed kidney paraffin block was sectioned at 4 μm and stained with periodic acid-Schiff (PAS). The level of renal injury was assessed on a blinded basis by calculating glomerular and tubulointerstitial injuries that we described previously [36]. Up to one hundred glomeruli were scored based on the 0 to 4+ injury scale to calculate the glomerular injury score. Tubulointerstitial injury (TI) scores were graded as absent (score 0), <10% involvement (score 1), 10–25% involvement (score 2), 25–50% involvement (score 3), 50–75% involvement (score 4), or 75–100% involvement (score 5). Placentas were sexed, resulting in four groups: control male offspring, CKD male offspring, control female offspring, and CKD female offspring. Five litters per group with three 3 μm sections per placenta were stained with hematoxylin and eosin and used for analysis. The cross-sectional area of the entire placenta was obtained, and placental zone analysis was performed using Image J [42]. The volumes of placental zones in each section were calculated as we described previously [38]. From these measurements, the ratios of the labyrinth zone, the junctional zone, and the decidua to whole placenta were calculated.

### 4.8. Statistical Analysis

Data are given as means ± SD. Statistical analysis was performed by Statistical Package for the Social Sciences (SPSS) software, version 16.0 (SPSS Inc.; Chicago, IL, USA). Parameters were compared using Student’s *t* test. A *p*-value < 0.05 was considered statistically significant.

## 5. Conclusions

Our findings highlight that maternal CKD poses not only adverse pregnancy outcomes but also increased risk for developing hypertension and kidney disease in adult offspring. Maternal adenine-induced CKD reshapes gut microbiota composition, mediates the microbiota-derived metabolite TMAO and SCFAs, increases uremic toxin ADMA and SDMA, and dysregulates the intrarenal RAS in offspring kidneys.

## Figures and Tables

**Table 1:** Plasma levels of nitric oxide (NO)- and trimethylamine-*N*-oxide (TMAO)-related parameters in mother rats.

□	Control	CKD
NO pathway		
L-citrulline, μM	56.2 ± 9.3	79.8 ± 20.4 *
L-arginine, μM	283.4 ± 29.1	190.7 ± 36.4 *
Asymmetric dimethylarginine, μM	1.59 ± 0.43	5.59 ± 1.54 *
Symmetric dimethylarginine, μM	0.76 ± 0.44	1.48 ± 0.26 *
L-arginine-to-asymmetric dimethylarginine, μM/μM	187.4 ± 44.9	35.6 ± 7.7 *
TMAO pathway		
TMAO, ng/mL	300.8 ± 44.9	1080.6 ± 162.1 *
Trimethylamine, ng/mL	198.7 ± 40.1	165.5 ± 41.5
Dimethylamine, ng/mL	233.5 ± 56.6	680.3 ± 125.8 *

CKD = Rats received chow supplemented with 0.5% adenine for 3 weeks before pregnancy; data are shown as means ± SD; *n* = 8/group; * *p* < 0.05 vs. control.

**Table 2:** Plasma and fecal levels of short chain fatty acids in mother rats.

□	Control	CKD
Plasma		
Acetate, μM	655.2 ± 297.7	413.9 ± 144.8 *
Propionate, μM	51.8 ± 15	50.4 ± 26.2
Butyrate, μM	52.8 ± 20	27.6 ± 12.4 *
Feces		
Acetate, mM/gm feces	5.91 ± 1.11	3.1 ± 0.68 *
Propionate, mM/gm feces	0.83 ± 0.35	0.38 ± 0.22 *
Butyrate, mM/gm feces	0.36 ± 0.23	0.24 ± 0.11

CKD = Rats received chow supplemented with 0.5% adenine for 3 weeks before pregnancy; data are shown as means ± SD; *n* = 8/group; * *p* < 0.05 vs. control.

**Table 3:** PCR primer sequences.

Gene	Forward	Reverse
*Ren*	5 aacattaccagggcaactttcact 3	5 acccccttcatggtgatctg 3
*Atp6ap2*	5 gaggcagtgaccctcaacat 3	5 ccctcctcacacaacaaggt 3
*Agt*	5 gcccaggtcgcgatgat 3	5 tgtacaagatgctgagtgaggcaa 3
*Ace*	5 caccggcaaggtctgctt 3	5 cttggcatagtttcgtgaggaa 3
*Ace2*	5 acccttcttacatcagccctactg 3	5 tgtccaaaacctaccccacatat 3
*Agtr1a*	5 gctgggcaacgagtttgtct 3	5 cagtccttcagctggatcttca 3
*Agtr1b*	5 caatctggctgtggctgactt 3	5 tgcacatcacaggtccaaaga 3
*Mas1*	5 catctctcctctcggctttgtg 3	5 cctcatccggaagcaaagg 3
*R18S*	5 gccgcggtaattccagctcca 3	5 cccgcccgctcccaagatc 3

## Abbreviations

ACE	Angiotensin converting enzyme
ACE2	Angiotensin converting enzyme 2
ADMA	Asymmetric dimethylarginine
ANOSIM	Analysis of Similarities
AT1R	Angiotensin II type 1 receptor
AT2R	Angiotensin II type 2 receptor
CKD	Chronic kidney disease
DMA	Dimethylamine
DOHaD	Developmental origins of health and disease
FID	Flame ionization detector
GD	Gestational day
LDA	Linear discriminant analysis
LEfSe	Linear discriminant analysis effect size
MAS1	Angiotensin (1–7) receptor
OTU	Operational taxonomic unit
PLS-DA	Partial Least Squares Discriminant Analysis
RAS	Renin-angiotensin system
SCFA	Short chain fatty acid
SD	Sprague–Dawley
SDMA	Symmetric dimethylarginine
TMA	Trimethylamine
TMAO	Trimethylamine-*N*-oxide
